# A Low-Power Spiking Neural Network Chip Based on a Compact LIF Neuron and Binary Exponential Charge Injector Synapse Circuits

**DOI:** 10.3390/s21134462

**Published:** 2021-06-29

**Authors:** Malik Summair Asghar, Saad Arslan, Hyungwon Kim

**Affiliations:** 1 Department of Electronics Engineering, Chungbuk National University, Chungdae-ro 1, Seowon-gu, Cheongju 28644, Korea; summair@chungbuk.ac.kr; 2 Department of Electrical and Computer Engineering, Abbottabad Campus, COMSATS University Islamabad, University Road, Tobe Camp, Abbottabad 22044, Pakistan; 3 Department of Electrical and Computer Engineering, COMSATS University Islamabad, Park Road, Tarlai Kalan, Islamabad 45550, Pakistan; saad.arslan@comsats.edu.pk

**Keywords:** spiking neural network, leaky integrate and fire, neuromorphic, artificial neural networks, artificial intelligence, image classification, CMOS

## Abstract

To realize a large-scale Spiking Neural Network (SNN) on hardware for mobile applications, area and power optimized electronic circuit design is critical. In this work, an area and power optimized hardware implementation of a large-scale SNN for real time IoT applications is presented. The analog Complementary Metal Oxide Semiconductor (CMOS) implementation incorporates neuron and synaptic circuits optimized for area and power consumption. The asynchronous neuronal circuits implemented benefit from higher energy efficiency and higher sensitivity. The proposed synapse circuit based on Binary Exponential Charge Injector (BECI) saves area and power consumption, and provides design scalability for higher resolutions. The SNN model implemented is optimized for 9 × 9 pixel input image and minimum bit-width weights that can satisfy target accuracy, occupies less area and power consumption. Moreover, the spiking neural network is replicated in full digital implementation for area and power comparisons. The SNN chip integrated from neuron and synapse circuits is capable of pattern recognition. The proposed SNN chip is fabricated using 180 nm CMOS process, which occupies a 3.6 mm^2^ chip core area, and achieves a classification accuracy of 94.66% for the MNIST dataset. The proposed SNN chip consumes an average power of 1.06 mW—20 times lower than the digital implementation.

## 1. Introduction

In recent years, neuromorphic systems, which are comparable to a biological neural network, have been widely investigated for prospective computing systems [1]. These neuromorphic systems consume very little energy and provide parallel signal processing [2]. Deep Neural Networks (DNN) are receiving much attention as the chosen classifier for various machine learning and computer vision applications due to their high classification accuracy [3]. However, for applications requiring a real environment using conventional von Neuman computing systems, the DNNs involve an immense number of computations and memory requirements. The bottleneck for DNN efficiency is the excessive and repeated update of data [4], high power consumption and memory bandwidth, making them not suitable for mobile applications where area and power are big constraints [5].

The driving force in field of computing has been to outperform the human brain using the von Neumann architecture. However, this architecture has significant differences with the human brain in terms of the organizational structure, computing methodology, area, and power constraints [6]. Today’s digital microprocessors have a basic architectural difference with the central nervous system. The von Neuman-based microprocessors, constituted of logic gates, have distinct computational and storage devices [7]. However, the latter is composed of a massive parallel arrangement of neurons, densely interconnected to one another in a vast network through links called synapses [8]. Moreover, in the central nervous system of the human brain, the processing elements (neurons) are placed very close to the memory units (synapses). The human brain emerges as a vast energy-efficient network by increasing the neuron–synapse interconnection, while consuming just 20 W and performing computations unmatched by modern computers [9]. Due to power and memory bottlenecks of conventional computers along with inspiration from biological achievements a new paradigm of neuromorphic architectures have evolved. Fundamentally, these are complementary to von Neumann architectures and have shown promising results in specific applications [10,11,12]. Neuromorphic architectures are not only energy efficient, but perform parallel signal processing, fault tolerance, and can be configurable. Moreover, they can be realized by numerous silicon-based technologies, large-scale architectures, and computational models of neural elements [13,14].

Although Artificial Neural Networks (ANN) can offer higher efficiency in training and inference tasks, they generally require higher complexity in hardware and power consumption than SNNs. Among various ANNs inspired from the human brain, Spiking Neural Networks aim to bridge the gap between neuroscience and machine learning. SNNs offer massive parallel computations on hardware, mimicking the human brain. SNNs process information via numerous neurons and communicate that information through a web of synapses. The transfer of information in the shape of small electric currents is carried out by a sequence of action potentials called spike trains. In light of the Shannon Theory, some studies suggest that the information contained in spike trains in the form of temporal codes are more energetically efficient than rate codes [15,16,17]. Neuromorphic architectures based on SNN benefit from computation organization—achieving high energy efficiency by co-locating computing (neurons) and memory (synapse) elements, and information representation—less power consumption by event-driven spikes encoding information [18]. The power constraints of mobile applications suffer due to the exponentially increasing processing complexity of large-scale ANN-based neuromorphic hardware and thus necessitate energy-efficient SNN-based neuromorphic hardware [19]. SNNs process the information by spike propagation, which enables it to accelerate the computational speed and improve energy efficiency [20]. It incorporates biologically plausible neuron models in acquiring the temporal dynamics of the neural membrane [21]. To realize the advantages of SNNs on a chip, it is crucial to optimize the neuron and synapse circuits for low power and a compact area. Therefore, to realize large-scale SNN hardware requires power and area optimized computational (neuron) and memory (synapse) elements.

When Carver Mead coined the idea of implementing neural networks in hardware, a new paradigm in neuromorphic engineering was established. The neural characteristics can be captured on hardware by the integration of robust and less power-hungry analog components [1]. Confronting challenges in the field of neuromorphic engineering requires a multidisciplinary approach, as the next generation VLSI technology can realize better hardware [22]. Implementing neuromorphic hardware requires numerous transistors to be integrated on the chip. This can be achieved to some extent by the scalability feat of the CMOS technology, although still incomparable with the integration density of the brain. Therefore, hybrid techniques that have merged conventional robust CMOS with newly developed technologies like memristors are attracting interest in research [23,24]. However, memristor crossbar architectures still require additional process requirements and are currently under study for simpler problems with discrete devices, whereas conventional CMOS, by virtue of its robustness and scalability has clear advantages for realizing large-scale neuromorphic hardware [25].

Since the emergence of neuromorphic architecture, several studies have been performed considering different large-scale architecture, technologies, and neuronal models. Neurogrid [26], a mixed signal and system, employs subthreshold circuits to model neuronal elements. This allows for a compact neuron area, but with all neurons sharing the same parameters and therefore becoming less configurable and power hungry. In BrainScaleS [21], a mixed signal approach was adopted where all the neurons have dedicated parameters. This allows high tunability, reconfigurability, and reliable parameter mapping at the cost of higher power consumption. The analog–digital neural network classifier [5] consists of an analog core with a current multiplier and environmental noise compensation circuits; it achieves area efficiency due to its recurrent operations. However, its excessive read and write operations via the digital controller makes it less energy efficient.

This paper presents the implementation of a large-scale SNN Artificial Intelligence (AI) hardware based on analog CMOS for real time IoT applications. The SNN hardware, optimized for area, power, and accuracy, is realized on a chip based on the preliminary prototype design [27]. The choice of SNN model is dictated by the analog design constraints for hardware. A four-layer fully connected SNN model is optimized for a reduced number of neurons, input image size, and weight resolution which can achieve targeted classification accuracy along with a minimal chip area. The proposed SNN comprises synapse and neuron circuits optimized for power and area using a 180 nm process design kit. Furthermore, a fully digital implementation of SNN is replicated to analyze and compare the power consumption and estimated chip area with the analog counterpart. This work elucidates our SNN model and design methodology, elemental circuit designs, simulations and calibrations, and measurement results from the test chip on board. Section 2 describes the overall SNN model architecture and design pre-considerations. In Section 3, the underlying analog CMOS circuits design and implementation is explained and validated by simulation results along with a description of its digital implementation. In Section 4, the measurements and performance result of analog and digital SNN are analyzed. Finally, a discussion and comparison of results achieved with other state-of-the-art large-scale neural networks is performed in Section 5, before concluding the paper in Section 6.

## 2. Architecture and Design

### 2.1. Spiking Neural Network Model

**Figure 1:**
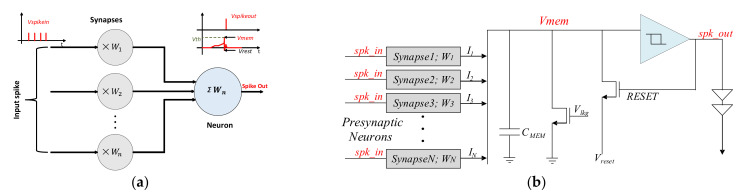
(**a**) Spiking Neural Network Model with a single neuron connected to multiple input synapses; (**b**) Leaky Integrate and Fire Model of the CMOS neuron cell.

The choice of neural network on chip for this work is SNN, regarded as the third-generation neural network, due to its biological feats and efficiency in the spatial–temporal signal coding [28]. In SNN, a web is weaved by the interconnections of neurons and synapses to perform inference and training tasks. Contrary to the continuous behavior of ANN, SNN works by making use of discrete events called spikes, which appear at specific time intervals. The occurrence of a spike event (0/1) mirrors the biological-chemical process of information delivery between different neurons. The neuron (post-synaptic) in a layer receives an input spike train from another neuron (pre-synaptic) in the previous layer, which are interconnected through synapses. The Figure 1a demonstrates a simple model of an SNN with a neuron, connected to a multitude of input synapses that receive input spike trains from pre-synaptic neurons. These input spike trains are modulated according to the respective synaptic strength (weight) and converted into current. The proportionate charge from all the synapses is then accumulated on the neuronal membrane as membrane potential. When membrane potential accumulates up to a predefined threshold value then the neuron emits or fires an output spike [29]. Thus, neurons act as an accumulation and comparison processing unit, while synapses are formed as memory with a communication interface.

### 2.2. Leaky Integrate and Fire Model

The choice of neuronal model for our large scale SNN is influenced by the pre-considerations of optimizing the design for area and power consumption, along with capturing the biological and temporal dynamics of the neuronal membrane. This requires that the neuronal model to be imitated replicates most of the computational studies, along with its simple design, which can unify a multitude of neurons on a chip. Therefore, for the implementation of large-scale SNN, our choice of model is the Leaky Integrate and Fire (LIF). This model possesses refractoriness and adaptation features to effectively mimic the biological computational features of neurons with good accuracy and adopts simpler circuit designs [30,31]. Contrary to other models [32,33], the LIF model for large scale neuromorphic architectures still draw the interest of researchers by virtue of the robustness of the CMOS design and its compact silicon implementation. Figure 1b models the LIF, where the neuron is represented by simple CMOS devices. The evolution of the neuronal membrane potential *V_mem_* can be modeled by a Resistor–Capacitor circuit, which is composed of a membrane capacitor *C_mem_* and a membrane resistance *R_mem_* (leakage path). A spiking neuron receives input spikes from the several pre-synaptic neurons interconnected via synapses. Each synapse acts a current source and injects a current equivalent to its strength called weights (*W*). The state (potential) of *C_mem_* is updated by injecting current through multiple current sources. Upon reaching a predefined threshold value, the comparator decides to evoke an output spike and resets the *V_mem_*.

The parallel combination of *C_mem_* and *R_mem_* can be defined by Kirchhoff’s current law [21] and is modeled by Equation (1). When an input current *I*(*t*) flows into a neuron, it will charge the *C_mem_* with *I_C_*(*t*) and discharge through the resistance with the current *I_R_*(*t*).

(1) $$I\left(t\right)=I_{R}\left(t\right)+I_{C}\left(t\right),$$

(2) $$I\left(t\right)=\frac{V_{mem}-V_{reset}}{R_{mem}}+C_{mem}\frac{\partial V_{mem}}{\partial t},$$

(3) $$C_{mem}\frac{\partial V_{mem}}{\partial t}=I\left(t\right)-\frac{V_{mem}-V_{reset}}{R_{mem}}.$$

When *V_mem_* ≥ *V_th_*, then *V_mem_* = *V_reset_*. In Equation (3) above, *V_mem_* is the membrane potential, *V_reset_* is the resting potential, and *V_th_* is the predefined threshold potential. When there is no input current, then the capacitive charge will decay by leaking through the resistance until it reaches the resting potential. In the resting state without any input current, the *V_mem_* stays at the resting potential *V_reset_*. In Equation (3), the *I*(*t*) is the summation of the excitatory and inhibitory input synaptic currents and is expressed as:

(4) $$I\left(t\right)=\underset{i}{\sum }\underset{f}{\sum }W_{i}\times I_{ref},$$

where *W_i_* denotes the weight strength of the *i*th synapse and ‘*f*’ is the number of the spike. *I_ref_* is the external reference current to be injected upon each input spike activity. To exhibit the inhibitory behavior of the synapse, the weights may have negative values, thus discharging the *I_ref_* upon each input spike activity.

## 3. SNN Implementation and Circuit Design

Learning in the neuromorphic systems can be categorized into on-chip [34,35] and off-chip learning [36,37]. The former mimics the biological neural systems, while the latter can benefit from the use of pretrained weights for achieving the same performance results as that of a software-based ANN [38]. Some SNN implementations employ their SNN architecture equivalent to an ANN, to determine trained weights using the ANN model in software and map the weights into the SNN hardware implementation [39]. Other works such as the STBP method in [40] have proposed direct training techniques that propagate spiking signals through the synapse and neuron models of the target SNN iteratively while converging the weights of each synapse for higher accuracy. Our proposed SNN architecture is based on the latter technique.

### 3.1. BSRC-Based SNN Architecture

For the SNN implementation proposed in this paper, the SNN model for the MNIST dataset is first optimized using a low-cost spike signal representation technique called Binary Streamed Rate Coding (BSRC), which was presented in our previous work [41]. While the proposed SNN employs the off-chip training technique, it determines the SNN’s floating point weights by propagating sequences of spikes through an accurate model of the target SNN chip, instead of an ANN counterpart as in most other SNNs. It then quantizes the floating-point weights into integer values of minimum bit-width while satisfying the target accuracy goal. In this paper, the target accuracy of 94% or higher is chosen, which leads to 4-bit quantized weights for our SNN chip implementation. The equivalence between our BSRC-based SNN model and its hardware implementation enables us to train the SNN with very high accuracy while allowing for a very small chip size.

**Figure 2:**
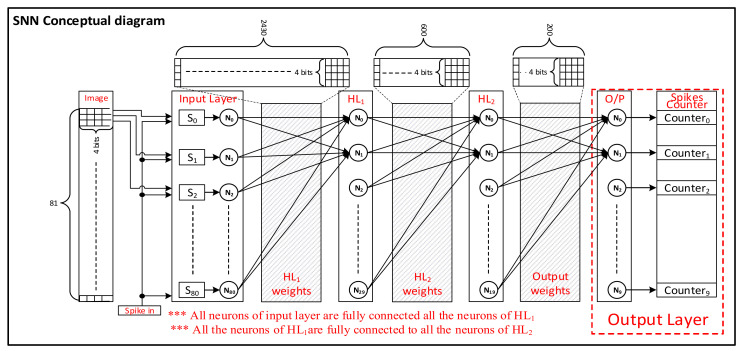
Block diagram of the SNN implementation consisting of four fully connected layers.

Figure 2 illustrates the overall block diagram for the proposed SNN hardware whose architecture is optimized using the BSRC-based SNN model. The SNN under study comprises four fully connected layers constituting synapses, neurons, and flipflops for weight and image memories. In order to minimize the chip size, our BSRC SNN optimization method reduced the input image size to 9 × 9 pixels from 28 × 28 pixels of the MNIST dataset. This proved to be the smallest size that could satisfy our target goal for accuracy of 94% or higher. The Input layer has 81 neurons for utilizing the 9 × 9 grayscale images as an input to the SNN. Our BSRC SNN optimization also determined the structure of each layer as follows: The output layer consists of 10 neurons for classifying the image, while two hidden layers consist of 30 and 20 neurons. All the neurons in each layer are fully connected with one another via 3311 synapses. After many iterations of the BSRC SNN optimization process, an SNN of four layers, namely (81-30-20-10), is determined, which consists of an input layer of 81 nodes, 1st hidden layer of 30 nodes, 2nd hidden layer of 20 nodes, and an output layer of 10 nodes.

The minimum weights are determined to be 4-bit wide, which are trained by a supervised training technique based on the BSRC-based backpropagation using the MNIST dataset. The 4-bits are stored in flip flop memories located close to the associated synapses in each layer, which leads to a short wire design, and consequently to faster operation and lower power consumption. The BSRC SNN optimization also chose 4-bits to represent the sequence of spike signals, which consists of a maximum of 15 spike pulses—no spike indicates a pixel value of zero, while 15 spikes denote a pixel value of 15. These spike pulses are provided to the inputs of synapse circuits in each layer, and also produced as outputs by neuron circuits of each layer. Each MNIST image is converted to an input spike sequence consisting of 15 possible spike pulses, proportional to the 4-bit pixel value of the image. In the output layer, a digital controller counts the number of spikes in each spike sequence and classifies the type of image on the measured spiking activity. The Analog CMOS-based implementation of the SNN’s constituent circuits and a fully digital implementation of SNNs are described in upcoming subsections.

### 3.2. Analog Spiking Neural Network

To realize a large-scale SNN on hardware requires not only the minimal SNN architectures, but also highly optimized CMOS circuits for minimal area and power consumption. The SNN model (81-30-20-10) in Figure 2 consist of 4 layers, where each layer has been built up from many unit cells that are constituted from Synapse and neuron circuits. These unit cells are designed from CMOS devices based upon the principles of the LIF model explained earlier. Keeping in mind the aforementioned design goals and constraints, a prototype synapse and neuron circuits were designed earlier [42], which can be integrated in the development of a large-scale SNN hardware while replicating most of the biological neuromorphic characteristics.

#### 3.2.1. Neuron Circuit

**Figure 3:**
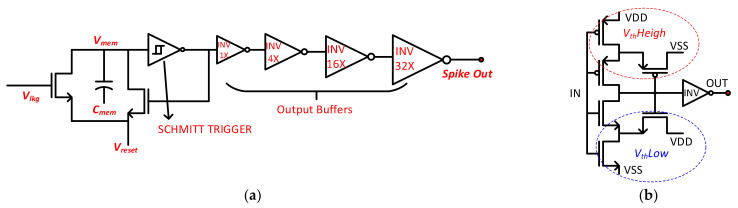
(**a**) The circuit schematic of the LIF-based neuron; (**b**) The circuit schematic of the Schmitt Trigger used inside the neuron circuit.

The LIF-based neuron circuit design is explained in Figure 3a. The neuronal membrane is simply modeled by a capacitor *C_mem_*, which integrates the synaptic currents in order to generate *V_mem_* across it. *C_mem_* is realized by the Metal–Insulator–Metal Capacitor (MIMCAP) to achieve better linearity along with less power consumption. The leakage path of the neuronal membrane is modeled by a resistor that is implemented via partially-on NMOS transistor to save the area. The decision of a neuron to fire an output spike is performed by a comparator for which we have implemented a Schmitt Trigger circuit as shown in Figure 3b. The Schmitt Trigger compares the *V_mem_* with the predefined threshold voltages. When the *V_mem_* exceeds the threshold voltage, the Schmitt Trigger fires an output spike, and the feedback path resets the membrane potential to the initial resting potential (*V_reset_*) via a separate NMOS transistor. As an important part of a neuron circuit, it needs careful designing in order to achieve optimum threshold voltages to ensure high speed and low power operations. Traditional neuron circuits often employ digital comparators requiring a global clock, which are power hungry as compared to asynchronous circuits. Analog asynchronous circuit implementations can achieve higher energy efficiency [43]. Therefore, the choice of asynchronous Schmitt Trigger for our neuron circuit provides a significant advantage by consuming less power and providing higher speed. Moreover, its simpler circuit implementation leads to small chip size, while its high sensitivity results in a highly accurate performance. The output buffers are implemented to provide the reshaping of the output spike pulses and isolation from the synapse circuits of the next layer.

#### 3.2.2. Synapse Circuit

**Figure 4:**
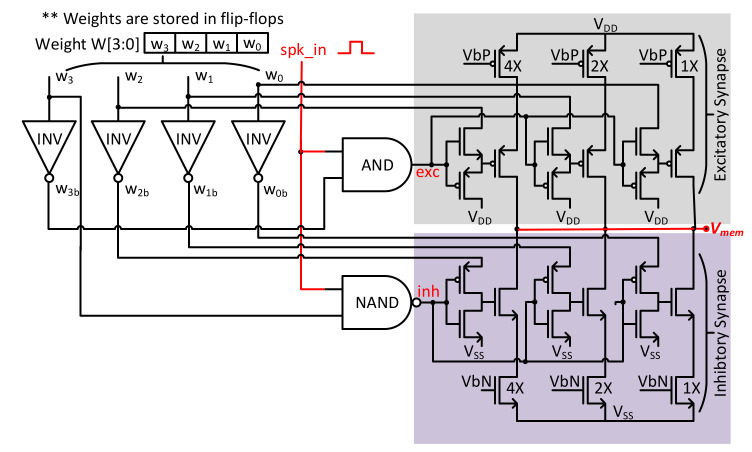
The circuit schematic for the BECI-based synapse with three branches and digital gates.

The synapse circuit for our SNN implementation is shown in Figure 4, which is based on a Binary Exponential Charge Injector structure and uses a 4-bit value of weight for each synapse. Each synapse circuit comprises an excitatory synapse and an inhibitory synapse. The excitatory synapse is composed of a pull-up PMOS network, while the inhibitory synapse consists of a pull-down NMOS network. Each network has 3 branches to realize the 3-bits of weight and are binary exponential sized for equivalent charge injection. The MSB of the 4-bit weight is used to switch between excitatory and inhibitory behavior through digital gates. If the MSB is 0, then the weight is positive and thus activates the excitatory synapse by injecting charge onto *C_mem_*. On the other hand, if the MSB is 1, then the weight is negative and thus activates the inhibitory synapse by discharging *C_mem_*. Upon receiving a spike event in each synapse branch, the weight of each synapse determines the amount of injected current from the excitatory synapse or the discharged current of the inhibitory synapse. The injected current of all branches is accumulated onto the membrane potential capacitor *C_mem_*. The BECI synapse circuit is inspired by the efficient structure of current mirroring DACs, which provides advantages including minimal number of transistors, minimum size for saving area, reduced power consumption, and design scalability for higher resolutions. For storing pretrained weight values on synapses, standard cell flip-flops collocated with each synapse circuit are used. Using this design method, not only can area be saved but energy required for read and write operations can also be reduced effectively.

#### 3.2.3. Circuit Simulation

The proposed SNN chip employs two types of synapse-neuron (one neuron cell) circuits: input layer circuit and hidden layer circuit. First, the input layer circuit is introduced, which consists of one synapse and one neuron to convert input pixels to spike sequences. Then, the hidden layer circuit comprising of multiple synapses and one neuron is described, which fires output spike signals based on weights and input spikes.

**Figure 5:**
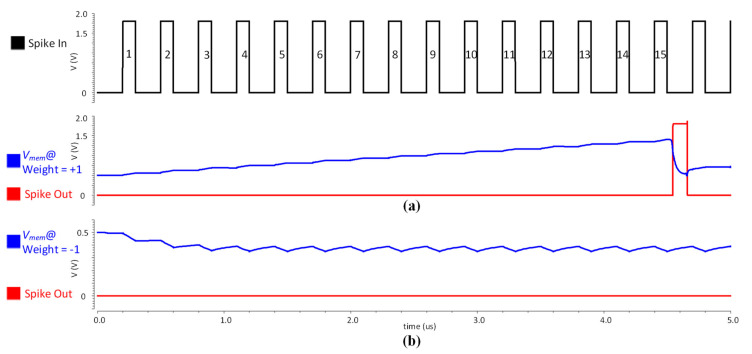
The circuit simulation results for one neuron cell of an input layer with an input spike train of 15 pulses: (**a**) shows the Vmem and spike-out when the weight value is +1; (**b**) shows the Vmem and spike-out when weight value is −1.

Figure 5 shows a circuit simulation result for one neuron cell of input layer, composed of a synapse and neuron circuits explained in the former sections. It verifies our BSRC spike generation mechanism upon receiving an input spiking event, which is designed based on the LIF model using 4-bit weight values. Upon receiving an input spiking signal, the BECI synapse circuit injects an amount of charge onto *V_mem_*, which is equivalent to its weight value. For this simulation, an input spike train acts as an initial enable signal, while the synapse is configured with both positive and negative weight values. Figure 5a shows an example of an excitatory behavior, where the synapse is configured with a positive weight value of +1, which slowly charges *V_mem_* with every input spike activity. Subsequently, the neuron fires an output spike when *V_mem_* reaches *V_th_*. Similarly, Figure 5b illustrates an example of an inhibitory behavior, where the synapse is configured with a weight value of −1, which discharges the *V_mem_* from its resting potential and the neuron refrains from firing an output spike. When the weight value is zero or when there is no input spike, then *V_mem_* discharges (leaks) towards *V_reset_* and the neuron does not fire an output spike.

**Figure 6:**
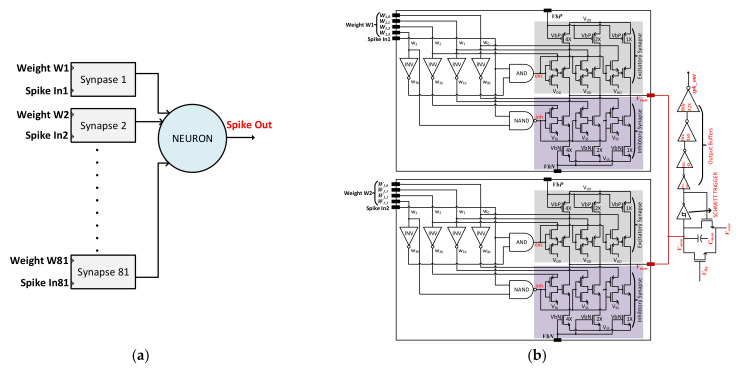
(**a**) Fully connected hidden layer with a large number of synapses connected to a single neuron; (**b**) One neuron cell of the hidden layers with two synapses connected to a single neuron.

Figure 6a illustrates the general structure of a hidden layer, while Figure 6b depicts a circuit diagram of an example of a hidden layer with two synapses and one neuron. Each synapse has its own distinguished weight value, which can be either excitatory or inhibitory. Due to large number of input synaptic activity, the neuron and synapses parameters need to be configured accordingly. To realize such a fully connected SNN, the circuit shown in Figure 6b was simulated. Here, two synapses with weight values W1 and W2 are connected to a single neuron constituting one neuron cell of hidden layers.

**Figure 7:**
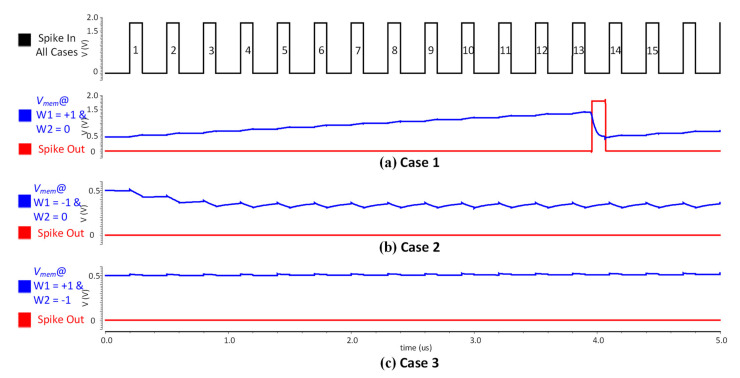
Circuit simulations for a neuron cell (one neuron connected to two synapses) where 15 input spikes are same for all the three cases: (**a**) shows Vmem and an output spike when weight values are W1 = +1 and W2 = 0; (**b**) shows Vmem and spike-out when weight values are W1 = −1 and W2 = 0; (**c**) shows Vmem and spike-out when weight values are W1 = +1 and W2 = −1.

Figure 7 shows the circuit simulation result for the hidden layer circuit given by Figure 6b. In the top graph of Figure 7, the spike-in signal of the current layer is the output spike signal from the previous layer. Although individual synapses receive their spike-in signals from different neurons’ outputs of the previous layer, in the simulation of Figure 7, for simplicity, the same spike-in signal is applied to the two synapses of Figure 6b. The synapses connected to a neuron in the hidden layer can have either positive or a negative weight value. The simulation verifies in three cases with two synapses connected to a neuron having a different combination of weight values. The first case in Figure 7a is when one synapse has a weight value W1 = +1, and the other has a weight value W2 = 0. This case demonstrates an excitatory behavior, where the neuron fires an output spike after receiving a sequence of input spikes. The second case illustrated in Figure 7b is when one synapse has W1 = −1 and the other has W2 = 0. It demonstrates an inhibitory behavior, where the neuron does not fire any output spike. The third case is depicted in Figure 7c, which shows the case when one synapse has W1 = +1 and the other has W2 = −1 weight value. Here the excitatory synapse injects the charge on *V_mem_*, while the inhibitory synapse discharges the *V_mem_* at the same amount. Therefore, *V_mem_* remains at resting potential *V_reset_* resulting in no output spiking activity. The simulation elucidates the biological neural behavior by the proposed circuit implementation of LIF model. The important simulation parameters along with neuron and synapse area and power estimation are summarized in Table 1.

### 3.3. Fully Digital Implementation of Spiking Neural Network

To evaluate the advantage of our analog implementation in terms of area and power, the BSRC-based SNN model is also implemented in a full digital design. The full digital design of SNN is verified on FPGA, while its area and power estimates are obtained using Synopsys Design Compiler using Standard Cell Library of the same process technology as the analog counterpart—TSMC 180 nm. The digital SNN realizes the same network shown in Figure 2 using Verilog HDL.

**Figure 8:**
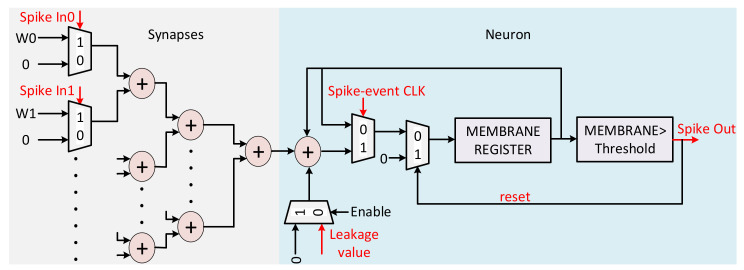
The block diagram of the full digital implementation for the output layer. A number of synapses accumulate in membrane register and the comparator fires the digital output spike pulses.

The input layer converts each input pixel value to a sequence of spikes using a design implementation of the integrate and fire (without leakage) neuron logic. The input layer converts 81 pixel values using 81 synapses in a way similar to the analog SNN implementation presented in Section 3.1. Figure 8 shows the detailed block diagram of a hidden layer implementation. Each neuron logic in the SNN has a membrane potential storage register and has a number of synapses attached as inputs. The neurons and synapses operate at discrete events represented by the rising edge of the spike-event clock. The spike-event clock is generated by dividing the system clock by an adjustable factor, which is set to 3 for the measurements results provided in Section 4.2. Each synapse, upon receiving a spike, adds its weight value to the membrane register. Whenever, the membrane potential exceeds the threshold value, the neuron generates an output spike and reset its membrane register value. The spikes are generated and received at the spike-event clock, while the width of each spike is equal to the system clock period.

## 4. Measurement Results and Analysis

This section analyzes the performance and measurements results of analog SNN and digital SNN implementations for determining an optimized hardware for the area and power constraints.

### 4.1. Implementation of Analog SNN

**Figure 9:**
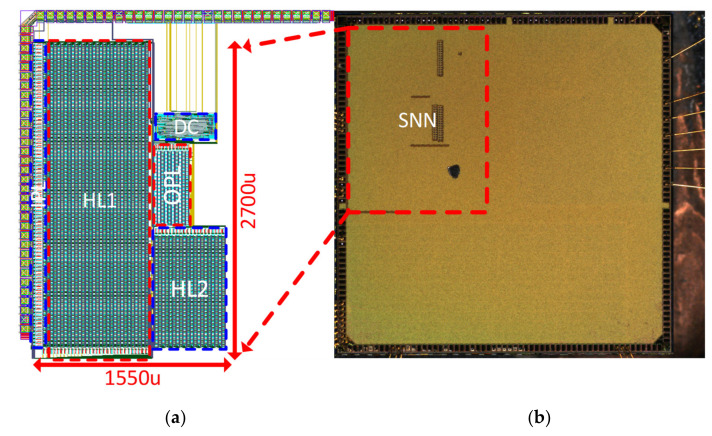
(**a**) The complete layout of the SNN implementation constituting of 4 fully connected layers with area estimation; (**b**) The bonded die micrograph highlighting the fabricated SNN chip.

The full-chip layout (including pads) of the analog SNN is described in Figure 9a. The test chip of the analog SNN is fabricated using the 180 nm CMOS process as part of a multiple project wafer. The fabricated chip’s micrograph with bonding wires is given in Figure 9b. The test chip occupies an active area of 3.6 mm^2^ within the allocated die area of 2700 μm × 1550 μm. The layout of the chip distinguishes the four fully connected layers of the SNN as input layer (IPL), hidden layer1 (HL1), hidden layer2 (HL2), output layer (OPL), and a Digital Controller (DC). The compactness is achieved via the tight placement of individual cells and smaller routing paths. The layers are placed side by side so that interconnection between two layers is of minimal length. Moreover, decoupling capacitors are added inside every neuron cell to allow for scalable and reliable design.

**Figure 10:**
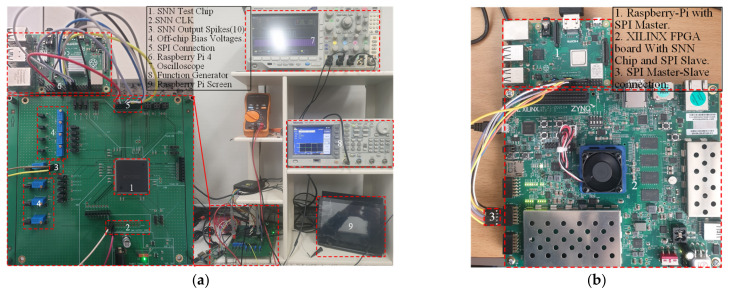
Measurement Setups: (**a**) analog SNN test chip mounted on test PCB is measured using oscilloscope, function generator, and a host CPU board (Raspberry Pi 4); (**b**) digital SNN implementation is measured via FPGA board interfaced with a host CPU board (Raspberry Pi 4).

Figure 10a shows a test printed circuit board (PCB) for testing the analog SNN chip and the measurement setup. A host CPU board (Raspberry Pi in our test setup) was used to download weight data and configuration parameters, and then provide input image to the SNN chip via a Serial Peripheral Interface (SPI). The on-chip digital controller activates the input layer to take pixel values from the image memory and convert them to input spike signals for SNN. Once all the spike signals are propagated through each layer, the output layer counts the final output spikes, while the digital controller sends back the data to the host CPU board for an evaluation of the classification results. Figure 10b shows the measurement setup for the fully digital implementation, which is described in Section 4.2.

**Figure 11:**
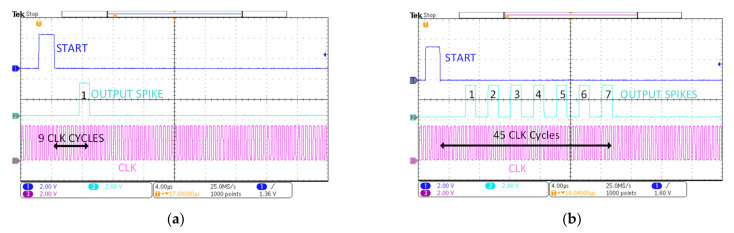
Measurement results for the spike propagation: (**a**) One input spike propagates through all layers; (**b**) seven input spikes propagates through all layers.

The measured results are shown in Figure 11. The spike propagation phenomenon is achieved successfully, as observed in circuit simulations. Figure 11a is the case when one input spike applied at the first neuron cell propagates through the first neuron cell of all the layers. This propagation is achieved by having maximum weight (7 in our implementation) for the first synapse of the first neuron in every layer, while all other weight values are set to zero. With one input spike applied to the first layer and the maximum weight value in every layer, the output layer generates one output spike. Similarly, in Figure 11b, 7 input spikes are applied to the first layer and propagated by using the same weight configuration as of Figure 11a. The measured results of Figure 11 verify the biological propagation of spikes from one layer to another layer for the SNN chip under test. The MNIST data set was applied during testing and the average on-chip current was measured for different applied images. The measured average current was 592 μW, which gives the power consumption of the test chip as 1.06 mW, while operating at a 10 MHz clock signal.

### 4.2. Implementation of Digital SNN

**Figure 12:**
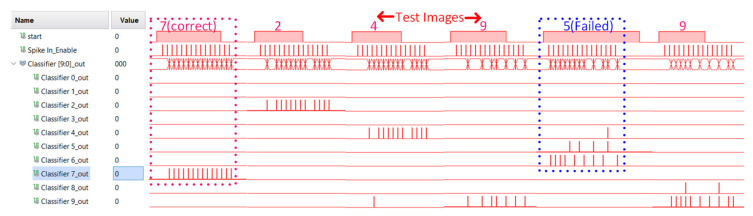
The measurement results for Digital SNN showing the correct classification of different input images along with the failed classification of input image ‘5’.

The aforementioned implementation of a fully digital SNN was tested using the measurement set-up described in Figure 10b. The fully digital SNN chip was implemented on an FPGA board (Xilinx ZYNQ Ultrascale), interfaced with Raspberry Pi 4 for the digital SNN measurements. Similar to analog SNN, the input image, weights data, and the configuration parameters for the digital controller are provided via SPI to the digital SNN. The controller generates stimulus input spike signals for SNN, counts the output spikes from the output layer, and sends back the data for further classification. The measured results obtained via Xilinx Integrated Logic Analyzer from FPGA are illustrated in Figure 12. The results show different input test images with respective to the classifier’s output spiking activity for correct and failed classification. For example, when applied with an input image of 7, the 7th node of the output layer exhibits the maximum spiking activity. The digital SNN has a measured area of 3.5 mm^2^ including routing overheads and a power consumption of 21.2 mW. This power consumption of the fully digital SNN chip was estimated by Synopsys Design compiler, while the power consumption of the analog SNN chip was measured from the fabricated chip. The estimated power consumption is accurate enough for our purpose of comparison, since the EDA tool was configured with an accurate 180 nm CMOS process PDK database provided by the same foundry.

Table 2 compares the area and power consumption between our analog and digital SNN implementations for the same optimized SNN model. The digital SNN implementation benefits from less design complexity and is easier to implement as compared to the CMOS-based analog SNN implementation. However, the analog SNN finds massive advantage over its digital counterpart in terms of power consumption while occupying nearly the equivalent area. This makes the analog SNN design a superior choice for a low-power implementation of SNNs for mobile applications.

## 5. Performance Analysis

**Figure 13:**
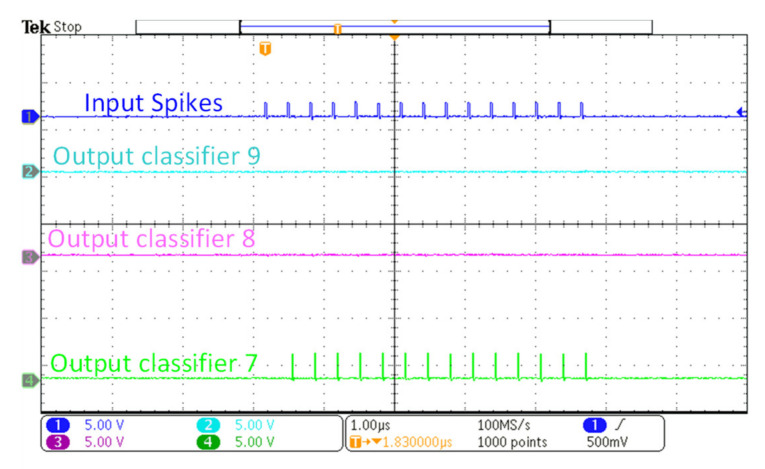
The measured results on the oscilloscope for the analog SNN. The applied input image to the SNN is ‘7’ and the 7th output classifier shows maximum spiking activity.

The classification analysis for implemented SNN is performed using MNIST—a handwritten digit dataset comprising of 10,000 test images. Firstly, an optimized SNN model is implemented in the Python framework considering its analog circuit architecture and chip size. Secondly, the SNN model is trained in the Python framework that follows the detailed architecture of the analog SNN structure. Finally, the trained weights are downloaded to the analog SNN chip to conduct the inference operation using the MNIST validation dataset. Figure 13 shows the case when the applied input image is ‘7’, the SNN predicts the correct classification result of ‘7’ by producing the maximum spiking activity at the 7th output node. The average classification accuracy achieved for the SNN is 94.66%, which is very close to the accuracy of 94.69% for the optimized SNN model in Python [41].

The performance of the SNN test chip has been compared with state-of-the-art works previously reported, as shown in Table 3. The comparison has been made among various neuromorphic chips to identify the optimum implementation in terms of area, power consumption, and classification accuracy. As the physical dynamics of various neuromorphic chips is different for diverse applications, the complexity of the system is therefore defined by the total number of weights. The complexity is then divided by total occupied area, and by the power consumed for the area, and the power efficiency (η) analysis, respectively. The large-scale biological plausible analog Neurogrid [26] implementation benefits from the compact neuron size (1800 μm^2^), which is based on the quadratic I & F model, achieves good area efficiency. Whereas our SNN based on the LIF model neuron cell occupies an almost similar area of 2022.72 μm^2^, it consumes much less power and achieves 40× higher power efficiency. The second generation analog BrainScaleS neuromorphic systems [21], comprised of neuronal array prototype chip with tunable parameters, has a compact area but is power hungry. The mixed-mode neural network classifier [5], based upon the Radial Biased Function Network (RBFN) and the Multilayer Perception (MLP), occupies less area due to its analog core implementation but is less power efficient. In contrast, the proposed SNN provides optimum area and power efficiencies and is thus suitable for AI mobile applications.

The energy consumption per spike was estimated for the current SNN chip by computing the total energy consumed for processing input spiking event divided by the number of the processed input spiking events [45]. As shown earlier in Figure 11a, for a single input spike it takes 9 clock cycles to evoke an output spike. It therefore consumes 900 pJ of energy per spike while operating at 10 MHz, which is different from energy per synaptic activation calculated as 941 pJ for [26]. The energy per spike for the proposed SNN is quite comparable with the 790 pJ energy per spike calculated for [21] and 900 pJ for [44]. Data for the accuracy was not available for the [26] and [21] analog implementations. Compared with the previous implementations [5], the proposed SNN implementation achieves an optimum classification accuracy at lower cost and power consumption, which makes it a strong candidate for mobile AI applications.

## 6. Conclusions

This work presents a hardware implementation of a large-scale SNN optimized for area and power, which is aimed at real-time AI/IoT applications. The SNNs allow for compact hardware implementation that is better suited for mobile or edge AI applications, if compact synapse and neuron circuits are used. Area and power efficient synapse and neuron circuits are proposed and an example SNN chip of 4 layers is constructed by integrating the synapse and neuron circuits. The SNN chip was implemented with a 180 nm CMOS process, which occupies a die area of 3.6 mm^2^ and consumes a power of around 1 mW. The analog SNN chip has an advantage over its digital counterpart in terms of power consumption while occupying almost same area. The SNN chip achieves a classification accuracy of 94.60%, which is comparable with its software model, while consuming 900 pJ of energy per spike, which is 20 times lower than the digital SSN chip. Moreover, the prototype SNN implementation can be easily expanded for higher resolutions and number of classes. As future work, we plan to develop a large-scale SNN chip that can be an alternative solution to ANNs for increased image size and number of classes.

## Figures and Tables

**Table 1:** Neuron Cell Simulation Specifications.

Parameters	Values
Neuron cell Area	2022.72 μm^2^
Neuron cell Power Consumption	25 μW
Resting potential	500 mV
Threshold Voltage	1.4 V
Resolution	4-bit
VDD/VSS	1.8 V/0 V
Membrane Capacitance	35 fF
Off-chip Bias voltages	8

**Table 2:** Comparison between the Proposed Analog SNN and Fully Digital SNN.

SNN Chip	Area	Power
Proposed Analog SNN Chip	3.6 mm^2^	1.06 mW
Fully Digital SNN Chip	3.5 mm^2^	21.2 mW

**Table 3:** Comparison of Performance of the SNN Chip with Other State of the Art Neuromorphic Chips.

Parameters	[26]	[21]	[5]	[44]	This Work
CMOS tech. [nm]	180	65	130	800	180
Architecture	Analog	Analog	Mixed-Mode	Mixed-Mode	Analog
Classifier type	SNN	SNN	MLP/RBFN	SNN	SNN
Neuron Model	Quad. I & F	LIF	Current mode	LIF	LIF
Chip Area [mm^2^]	168	3.6	0.140	1.6	3.6
Power [mW]	3100	48.62	2.20	40 μ ^1^	1.06
Energy/Spike [pJ]	941 ^2^	790	-	900	900
Accuracy (%)	-	-	92	-	94.60
Complexity [Total # of weights]	256 K	1024	750	256	3311
Area η [Complexity/Area]	1523	284.5	5360	160	920
Power η [Complexity/power]	82.5	21.06	341	-	3123

^1^ For one neuron. ^2^ Energy per synaptic operation.

## Data Availability

Not applicable.
